# Development and Validation of Analytical Methods for Radiochemical Purity of ^177^Lu-PSMA-1

**DOI:** 10.3390/ph15050522

**Published:** 2022-04-24

**Authors:** Pauline Orhon, Marie-Dominique Desruet, Marie Piquemal, Nicolas De Leiris, Loïc Djaileb, Jean-Philippe Vuillez, Pierrick Bedouch, Julien Leenhardt

**Affiliations:** 1 Pharmacy Department, Grenoble Alpes University Hospital, 38000 Grenoble, France; mddesruet@chu-grenoble.fr (M.-D.D.); marie.piquemal@chu-lyon.fr (M.P.); pbedouch@chu-grenoble.fr (P.B.); jleenhardt@chu-grenoble.fr (J.L.); 2 LRB, CHU Grenoble Alpes, INSERM, Grenoble Alpes University, 38000 Grenoble, France; ndeleiris@chu-grenoble.fr (N.D.L.); ldjaileb@chu-grenoble.fr (L.D.); jpvuillez@chu-grenoble.fr (J.-P.V.); 3 Nuclear Medicine, Grenoble Alpes University Hospital, 38000 Grenoble, France; 4 TIMC-IMAG, UMR 5525, CNRS, Grenoble Alpes University, 38000 Grenoble, France

**Keywords:** radioligand therapy, prostate cancer, radiochemical purity, ^177^Lu-PSMA-1, validation of analytical methods, high-performance liquid chromatography (HPLC), thin-layer chromatography (TLC)

## Abstract

Prostate Specific Membrane Antigen (PSMA) is a highly relevant target in nuclear medicine due to its overexpression in prostate cancer. The ^68^Ga/^177^Lu-PSMA-1 combination is a theranostic agent for the detection and treatment of tumors overexpressing the PSMA target. Specifically, ^177^Lu-PSMA-1 is used in the treatment of castration-resistant prostate cancer that is ineffective or intolerant to the latest generation of chemotherapy and/or hormone therapy. This radiopharmaceutical is manufactured in a radiopharmaceutical synthesizing unit and must pass a quality control where the radiochemical purity (RCP) is assessed prior to release of the batch. RCP evaluation is performed by high-performance liquid chromatography (HPLC) and thin-layer chromatography (TLC). Since there is no monograph for ^177^Lu-PSMA-1 in the European Pharmacopoeia, we validate the analytical methods according to the EANM recommendations adapted from ICH Q2. Specificity, linearity, accuracy, precision, intermediate precision, limit of quantification (LOQ) and robustness were described for HPLC and TLC in this study. The results obtained demonstrated the robustness and reliability of the HPLC and TLC analytical methods for the evaluation of the RCP of ^177^Lu-PSMA-1.

## 1. Introduction

Prostate cancer (PC) is the leading men cancer, ahead of lung and colorectal cancer. It represents 25% of all incidents of male cancers. Across the globe, in 2018, 1.3 million new cases were found, with 50,400 in France [1]. Approximately 66% of prostate cancer cases occur in men aged 65 and over. Median age at diagnosis is 68 years. Occasional before 50 years, its incidence increases progressively with age. It is the third most common cancer death for men. Nearly 79% of deaths affect men around 75 years, and 359,000 deaths were estimated in 2018 worldwide [2], with 8100 in France. Patients with localized PC are especially treated by surgery with radical prostatectomy or radiotherapy [3]. For metastatic PC, treatments are hormonotherapy (first and second generation) and chemotherapy [4,5].

PSMA is a cell surface glycoprotein overexpressed on prostate cancer cells (approximately 1000 times more in PC cells than in normal epithelial cells). Its expression increases with tumor level and Gleason score, in locally advanced tumors and in castration-resistant forms [6,7]. High-affinity small molecule PSMA ligands enable whole-body tumor-specific imaging (^68^Ga-PSMA PET/CT) and systemic targeted therapy (^177^Lu-PSMA) [8,9,10,11]. 

^177^Lu-PSMA-1 radioligand therapy (RLT) is a therapy using a PSMA-1 ligand labeled with ^177^Lutetium indicated for the treatment of metastasized castration-resistant prostate cancer (mCRPC) [12,13]. In France, this RLT is now available with a temporary authorization. Eligibility criteria are a progressive disease after chemotherapy, a second-generation hormonotherapy (abiraterone or enzalutamide) and a high positive PSMA PET/CT uptake. In order to be authorized to carry out this type of treatment, French radiopharmacies must draft and submit an Investigational Medicinal Product Dossier (IMPD) to the national agency for medicines and health products. Part of this IMPD is intended for analytical methods validation in order to determine the radiochemical purity (RCP) by high-performance liquid chromatography (HPLC) and thin-layer chromatography (TLC) methods since there are currently no monographs available in the European or French pharmacopoeias. The aim of this study is to describe and validate the analytical methods for determining the radiochemical purity of the radiotracer ^177^Lu-PSMA-1 by HPLC and TLC according to the guidelines of the European Association of Nuclear Medicine (EANM) [14,15].

## 2. Results

### 2.1. Validation of the Analytical Method: Radiochemical Identity

**Figure 1:**
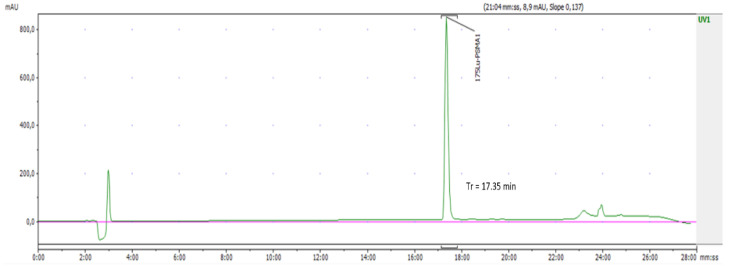
Retention time of the ^175^Lu-PSMA I/T standard.

**Figure 2:**
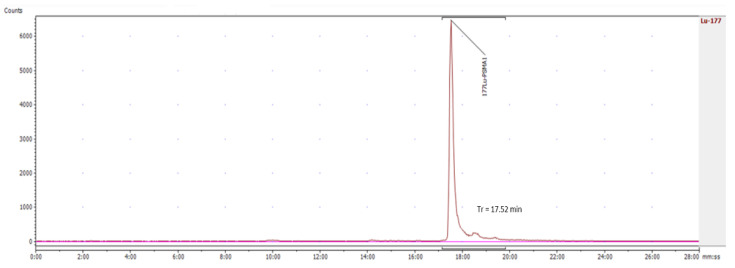
Retention time of ^177^Lu-PSMA-1 complex.

The radiochemical identity test confirmed that the retention time (Rt) of the main peak of the radioactive product corresponded to the retention time of the non-radioactive reference standard. Under HPLC analytical conditions, the deviation between the two retention times was 0.17 min (Figure 1 and Figure 2). This test was only available via HPLC analytical method. We injected different concentrations of ^175^Lu-PSMA I/T standard, and the mean area under the curve values as well as the mean retention times were collected. These results are attached in the Supplementary Materials (Table S1). The straight linear regression equation between the concentration of the standard and the peak area permitted us to calculate the linear correlation coefficient, which was 0.998 (Supplementary Materials, Figure S1).

### 2.2. Validation of the Analytical Method (HPLC and TLC): Radiochemical Purity

#### 2.2.1. Specificity

- HPLC method:

The efficiency of the column was correlated to the plate count (N) and the tailing factor (TF). In the HPLC analytical method, the mean of N was 6725.70 ± 153.60 (mean ± standard deviation) and the TF was 1.25 ± 0.08 (Table 1). These results were in agreement with the acceptance criteria (N > 6000 and TF between 0.8 and 1.3) [16].

Specificity of the analytical method was evaluated by comparing the retention times of ^177^Lu-PSMA-1 (radiopharmaceutical) and free ^177^Lu (impurity) and determining the resolution (Rs). Rs between the ^177^Lu impurity and the ^177^Lu-PSMA-1 peak was 17.73 (Table 1). This resolution value was significantly higher than the reference value. Therefore, this analytical method was specific to correctly separate potential impurities from the ^177^Lu-PSMA-1 radiopharmaceutical.

- TLC method:

For the TLC method, the resolution (Rs) between the ^177^Lu impurity and the ^177^Lu-PSMA-1 peak was 3.060 (Table 2). This result was in agreement with the acceptance criteria (Rs > 2).

#### 2.2.2. Accuracy

- HPLC method:

The recovery percentage was calculated for each proportion of ^177^Lu-PSMA-1. The range of these values was 100.00% to 101.67%, which was consistent with acceptable values (90–110%) (Table 3).

- TLC method:

For the TLC detector, the range of recovery percentage values was 98.47% to 100.67%, which complied with the specifications (90–110%) (Table 4).

#### 2.2.3. Precision: Repeatability and Intermediate Precision

- HPLC method:

The precision of the radio detector was determined by the repeatability and the intermediate precision. The results obtained for retention time and RCP were consistent with variation coefficients lower than 2% (Table 5).

- TLC method:

The precision of the radio detector was determined by the repeatability and the intermediate precision. The results achieved were developed in Table 6: we obtained a variation coefficient lower than 2% for the RCP and retardation factor, except for the RSD of the retardation time of the 3-day series (RSD = 2.99%).

#### 2.2.4. Linearity

The linearity of the HPLC and TLC radio detector was assessed by integrating the area under the curve (AUC). The AUC represented the number of counts per second (cps) of the main substance peak (^177^Lu-PSMA-1) according to the activity injected by HPLC or TLC obtained by different dilutions of a stock solution.

**Figure 3:**
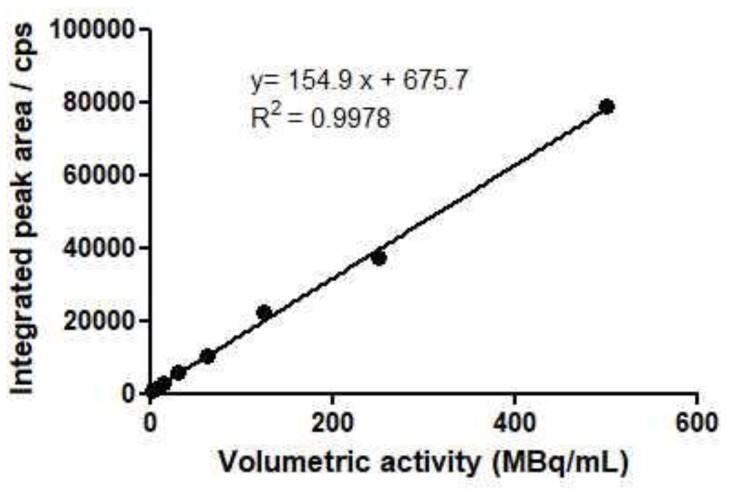
Linearity in HPLC method.

- HPLC method:

The equation of the regression line allowed us to calculate a linear correlation coefficient (R^2^) of 0.9978. This confirmed the linearity of the method, since R^2^ met the acceptance criteria (>0.99) (Figure 3)**.**

**Figure 4:**
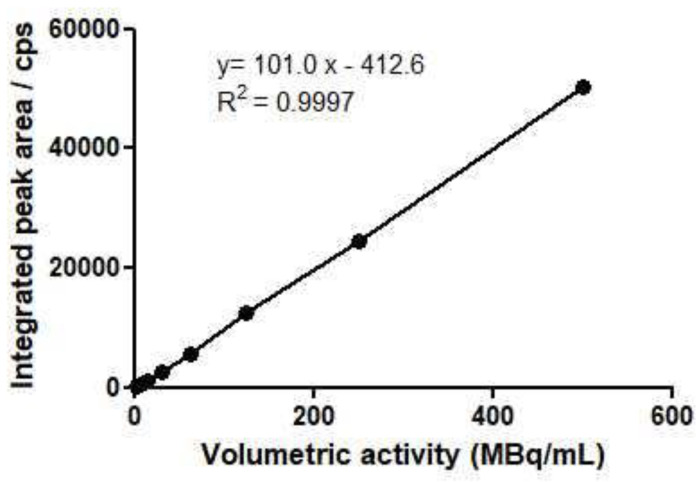
Linearity in TLC method.

- TLC method:

In this example (Figure 4), the equation of the regression line allowed us to calculate the linear correlation coefficient, which was 0.9997. Linearity was demonstrated.

The linearity could also be assessed by the correlation of the recovered RCP and the theoretical RCP of ^177^Lu-PSMA-1 by collecting the accuracy data. These calibration curves are attached in the Supplementary Materials (Figures S2 and S3).

#### 2.2.5. Robustness

- HPLC method:

Once we modified the HPLC flow rate (condition A), we noticed the decrease of the ^177^Lu-PSMA-1 retention time with a mean of 13.24 min ± 0.08 min and an RSD of 1.07%. This result was consistent with the increased flow rate of the HPLC. Therefore, the radiotracer peak tended to emerge more quickly. The RCP was similar to other measures determined under conventional analytical conditions: 98.45 ± 0.06 with RSD of 0.06%. 

By decreasing the percentage of trifluoroacetic acid of the HPLC’s mobile phase (condition B), we noticed a slight increase of the retention time—17.53 min ± 0.03 min—and an RSD of 0.28%. The RCP was similar to other measures determined under conventional analytical conditions: 98.72 ± 0.30 with a RSD of 0.30%.

- TLC method:

When we changed the mobile phase of the TLC system (condition B: 100% of 1 M ammonium acetate), we observed a decrease of ^177^Lu-PSMA-1 retardation factor 0.37 ± 0.04 vs. 0.92. The peak obtained was more crushed. The RCP remained equivalent to 98.55 ± 0.50 with an RSD of 0.51%.

The results were unusable for condition A with 100% of Methanol solution. 

#### 2.2.6. Quantification Limit (LoQ)

The limit of quantification of the radio detector was an important parameter of our analytical method, as the expected activity values of impurities were very low. It was therefore necessary to know from which value the radio detector allowed us to quantify a signal as precisely as possible. We measured the limit of quantification by performing dilutions of a sample to obtain a signal-to-noise HPLC or TLC > 10. Under these conditions, we obtained the following values:

- For the HPLC method: LoQ = 33.8 kBq (Signal/Noise = 12.7:1).
- For the TLC method: LoQ = 61.8 kBq (Signal/Noise = 10.8:1).

#### 2.2.7. Range

The applicable radioactivity range for the samples of ^177^Lu-PSMA-1 was calculated thanks to the product specifications. The maximum activity provided was 7400 MBq in 15 mL. This equated to a radioactivity concentration of 493 MBq/mL. The upper range of the linearity test was 500 MBq/mL. The lower range limit derived from the LOQ based on a minimum detectable ^177^Lu content of 0.5% [15]. In order to meet this requirement, the applied radioactivity concentration was 73 times higher than the LOQ, i.e., 73 × 33.8 = 2.5 MBq/mL, for HPLC method and 40 times higher than the LOQ, i.e., 40 × 61.8 = 2.5 MBq/mL, for TLC method. This equated to 37.5 MBq in 15 mL. The validated analysis range was from 2.5 to 500 MBq/mL for HPLC and TLC method.

## 3. Discussion

This manuscript describes the validation of an HPLC and TLC method to determine the radiochemical purity of the radiopharmaceutical ^177^Lu-PSMA-1. Currently, there is no monograph for ^177^Lu-PSMA-1 in the European pharmacopoeia. Therefore, we need to validate our analytical methods in order to demonstrate that the procedure is suitable for the assessment of radiochemical purity of ^177^Lu-PSMA-1. Following the recommendations of the EANM and the European Pharmacopoeia, we validated the parameters of specificity, accuracy, linearity, precision with repeatability and intermediate precision, limit of quantification, robustness and range for each of the two methods. This publication details the first study validating a dual analytical method using HPLC and TLC to determine the RCP of a radiopharmaceutical used in nuclear medicine therapy [15,17]. Many studies have already been published on the validation of analytical methods applied to radiopharmacy, in particular on the assessment of the radiochemical purity of specific radiopharmaceuticals. However, to date, none of these studies have combined an evaluation by HPLC and TLC [15,16,18,19,20,21,22,23,24,25].

Usually, the quality controls used to evaluate the RCP of radiopharmaceuticals commonly employed in nuclear medicine are performed with the TLC method, which provides a faster result. However, when developing new radiotracers and especially for ^177^Lu-labeled radiopharmaceuticals, the guidelines recommend including a quality control by HPLC in addition to those performed by TLC. The HPLC method provides two chromatograms via a UV detector and a radio detector (often coupled in series). The chemical purity of a radiotracer can be achieved via the UV detector. In our study concerning ^177^Lu-PSMA-1, the chemical purity could not be obtained because the UV signal corresponding to the ascorbate reagent was too strong, overwhelming the other signals. Therefore, in this study, we focus on the analytical validation of HPLC and TLC for the determination of the radiochemical purity. The advantage of coupling HPLC with TLC to determine radiochemical purity lies in the fact that HPLC may irreversibly adsorb some compounds and make them invisible on the chromatogram. It is therefore more appropriate to use two independent chromatographic methods to verify the radiochemical purity.

Table 7 is a summary of the results obtained for the HPLC and the TLC methods concerning the analytical validation of the RCP of ^177^Lu-PSMA-1. The results obtained are consistent with the specifications except for the intermediate precision concerning the retardation factor in TLC. This can be explained by a non-reproducible migration time of the strip or different strip sizes. However, this does not affect the evaluation of the RCP because the specificity for each analysis was optimal (Rs > 2). We therefore decided to validate these results.

In this manuscript, we focused on the validation of the analytical method to assess the radiochemical purity of ^177^Lu-PSMA-1. Other criteria also need to be validated before the release of a production batch, such as the evaluation of radionuclide purity, which is an important requirement to consider.

## 4. Materials and Methods

The investigational drug (ID) was a peptide ligand: PSMA-1 radiolabeled with ^177^Lutetium.

### 4.1. Reagents and Equipment 

Ascorbate buffer composed of ascorbic acid and sodium ascorbate, ITM-PSMA (Isotope Technologies Munich) peptide composed of 115μg of powder and purified water were obtained from ITM. Lutetium-177 (EndolucinBeta^®^) was received from Iason GmbH (Graz, Austria). Injectable solution of sodium chloride 0.9% (50 mL bottle) was obtained from Fresenius Kabi (Bad Homburg, Germany). ^175^Lu-PSMA I/T was used as reference standard for ^177^Lu-PSMA-1. It was purchased from ABX. ^175^Lu-PSMA-I/T was prepared as mentioned below. Free ^177^Lu considered as a radiochemical impurity was purchased from Iason GmbH.

Analytical HPLC was carried out using column eluting with a pump and UV detector Shimatzu LC-20AD (Kyoto, Japan) coupled with the radio detector Flow-Ram (Lablogic, Brandon, FL, USA) and controlled by the software Laura (edition 4 sp1, Lablogic, Brandon, FL, USA). Flow-Ram is a NaI detector, and the parameters for this analytical method were as follows: 850 V for photomultiplier voltage and tubing of 0.018 cm3 for liquid scintillation flow cell volume.

HPLC was equipped with an Agilent C18 column 5μm (150 × 4.6 mm). For the detection of chemical impurities, the samples were monitored with a UV detector at 220 nm and the column temperature was around 20 °C. The used solvents were (A) ultra-pure water + 0.1% trifluoroacetic acid (TFA) and (B) acetonitrile + 0.1% TFA. TFA was purchased from Sigma Aldrich (HPLC > 99%) and acetonitrile from VWR (HPLC—super gradient Reagent Eur. Ph). The flow rate of the mobile phase was set at 0.6 mL/min, with a total run of 28 min. The following gradient was used upon HPLC analysis: 0–17 min 15% B to 32% B, 17–17.5 min 32% B to 60% B, 17.5–20.5 min 60% B, 20.5–21 min 60% B to 15% B and 21–28 min 15% B.

Analytical TLC was carried out using the radio detector Scan-Ram (Lablogic, Brandon, FL, USA) and ITLC SG strips (1 × 10 mm). Scan-Ram is a plastic scintillation detector, and the parameters for this analytical method were as follows: 820 V for photomultiplier voltage and collimator of 3 mm. The mobile phase was composed of 1 M ammonium acetate and methanol (1:1). ^177^Lu-PSMA-1 migrates with the solvent, i.e., a retardation factor (Rf) between 0.7 and 1.0. Free ^177^Lu remains at the baseline, i.e., an Rf between 0.0 and 0.3. Methanol was obtained from VWR (Reagent Eur. Ph for HPLC) and ammonium acetate from Carlo Erba (reagent for analysis).

### 4.2. Synthesis Development

The radiotracer was produced in a specific radiopharmacy laboratory in a class A high-energy shielded enclosure. Radiolabeling was carried out in a closed system using the miniAIO^®®^ synthesis device (Trasis, Ans, Belgium).

Firstly, we prepared the raw materials in the leaded enclosure. We needed to reconstitute the ascorbate buffer with 2 mL of water (we used a syringe and needle provided by ITM). Then, we transferred the reconstituted ascorbate buffer into the ITM-PSMA peptide vial. Subsequently, we turned on Trasis software and placed the ^177^Lu-peptide cassette on the automaton. Fourthly, we connected the reconstituted peptide vial in the second position of the cassette, the 0.9% NaCl vial in position 5 (via the spike) and the EndolucinBeta^®®^ vial in position 1. Finally, we started the synthesis via the Trasis software controlling the device. The reconstituted peptide with ascorbate buffer was transferred to the EndolucinBeta^®®^ precursor vial. Then, it was transferred to the reaction vial to be heated at 95 °C for 20 min. A cooling step was performed by removing the reaction vial from the reactor by placing it in a leaded vial to let it cool at room temperature for 15 min. Finally, the last stage allowed for the sterilizing filtration and the formulation of the final product.

### 4.3. Validation of the Analytical Method—Radiochemical Identity 

Validation of ^177^Lu-PSMA-1 radiochemical identification by HPLC was performed by determining the retention time of the main peak of the radioactive product, which must correspond to the retention time of the non-radioactive reference standard. It must be within ±5%. The delay time between the UV and the radioactivity detector was taken into account. It represented 0.1 min. ^175^Lu-PSMA-I/T standard solutions were prepared at various concentration levels ranging from 15 µg/mL to 500 µg/mL. 

Although not recommended by the EANM for the radiochemical identity, we decided to evaluate the precision/repeatability and linearity of our HPLC analytical method with the “cold” radiotracer. For this, ^175^Lu-PSMA-I/T standard solution was injected at five different concentrations, three times. For each concentration, the average of the integrated peak areas as well as its standard deviation (SD) were calculated using Microsoft Excel 2013. From these values, the relative standard deviation was determined according to the formula: RSD (%) = (SD/mean) × 100. RSD has to be less than 2% [16,18]. The calibration curve was performed with GraphPad Prism 5 software by linear regression analysis of the average integrated peak areas versus the known concentration of standard solution. The correlation coefficient (R^2^) should be greater than 0.99. 

### 4.4. Validation of the Analytical Method—Radiochemical Purity 

Validation of the analytical method for the determination of RCP of ^177^Lu-PSMA-1 was carried out according to ICH Q2 guidelines [26] and EANM guidelines [15]. In this context, a validation of the following parameters was necessary.

Means, standard deviations and variation coefficients (RSD %) of the RCP, as well as the retention time (Rt) and the retardation factor (Rf) were calculated, and linear regression coefficients (R^2^) were determined for linearity and accuracy evaluations. 

#### 4.4.1. Specificity 

Specificity allowed us to verify the ability of the analytical method to obtain an unequivocal evaluation of the active substance in the presence of other critical compounds that could be found in the final product (impurities for example).

For this purpose, we evaluated the resolution, the retention times of the main substance and the impurities. We injected the potential radioactive impurity, i.e., free ^177^Lu, and the radiopharmaceutical ^177^Lu-PSMA-1 three times. The specificity of our analytical method was evaluated by comparing the retention times of the different entities and calculating the resolution by the resolution factor (Rs) with the following formula: Rs = $2\frac{Tr2\;-\;Tr1}{W2\;+\;W1}$. Rs should be greater than 2 (Tr represented the retention time of the components and W the peak width to baseline). 

Furthermore, to evaluate the efficiency of the HPLC analytical system, we measured the retention time, the area under the curve (AUC) of the peak, the tailing factor (TF) and the plate count (N).

#### 4.4.2. Accuracy

Accuracy was used as a measure of the degree of conformity of a value generated by a specific procedure. The relative error was the difference between the value obtained and the reference value. For this purpose, we investigated that for different mixtures composed of the main substance (^177^Lu-PSMA-1) and an impurity (free ^177^Lu) at different known concentrations, and a proportionality between the areas of the peaks was found. We prepared two solutions of ^177^Lu-PSMA-1 and free ^177^Lu with the same volumetric activity (180 MBq/mL), and we mixed these two solutions in different proportions in order to obtain the following concentrations:

- 100% of main solution or 50 µL of ^177^Lu-PSMA-1;
- 90% of main solution, i.e., 45 µL of ^177^Lu-PSMA-1 and 5 µL of free ^177^Lu;
- 80% of main solution, i.e., 40 µL of ^177^Lu-PSMA-1 and 10 µL of free ^177^Lu;
- 70% of main solution, i.e., 35 µL of ^177^Lu-PSMA-1 and 15 µL of free ^177^Lu;
- 60% of main solution, i.e., 30 µL of ^177^Lu-PSMA-1 and 20 µL of free ^177^Lu.

We determined the recovery percentage with the following formula: % recovery = $\frac{\left(\mathrm{RCP}\;\mathrm{recovered}\right)}{\left(\mathrm{RCP}\;\mathrm{Theoretical}\right)}\times 100\;$. RCP theoretical = $\frac{\mathrm{RCP}\;\mathrm{original}\;*\;\mathrm{target}\;\mathrm{percentage}}{100}$ (RCP original is the radiochemical purity of the original sample, and RCP recovered is the radiochemical purity of the mixed solution). The considered acceptable recovery criteria was in the 90–110% range [15].

#### 4.4.3. Precision, Repeatability and Intermediate Precision

Precision of the analysis method was determined based on repeatability and intermediate precision. The precision allowed us to express the fidelity evaluated under identical operating conditions and in a short time interval. It revealed the closeness of the results when repeatedly measuring a homogenous sample with the same analytical method.

For the determination of the repeatability of the method, we used six radiochromatograms from one synthesis of ^177^Lu-PSMA-1. We integrated the peaks corresponding to ^177^Lu-PSMA-1 and impurities, in order to obtain the retention times (Rt) and RCP. Statistical analysis was used to obtain the mean, standard deviation and variation coefficient values. 

The intermediate precision allowed us to evaluate the influence that random phenomena can have on the precision of our method. For this purpose, we analyzed the radiochromatograms of nine production batches produced by three different people, on nine different days. Statistical analysis was used to obtain the mean, standard deviation and variation coefficient values for retention times and RCP. 

#### 4.4.4. Linearity

Linearity allowed us to verify that for different concentrations of the active substance, and the response of the device was linear. The statistical function used was the linear regression with the least squares method. The equation of the curve and the correlation coefficient were calculated according to the equation of y = ax + b, where

y was the concentration of our sample or the number of counts,
a was the slope of the line,
x was the area under the curve of the active substance peak
and b was the intersection of the line with the *y*-axis.

As the decay of our radionuclide was long (6.7 days), we performed dilutions of the final product (^177^Lu-PSMA-1), which were injected in triplicate into HPLC or spotted on a strip for TLC method (5 mL on TLC strips). We were able to calculate the correlation coefficient (R^2^), which must be greater than 0.99.

#### 4.4.5. Robustness

The robustness of the method was its ability to withstand small deliberate variations in parameters. This provided an indication of its reliability under normal conditions of use. To evaluate this parameter in HPLC, we decided to modify the elution rate and the pH of the mobile phases. For TLC, we modified the composition of the mobile phase.

Two conditions were listed below for the HPLC method:

- Condition A: increase HPLC flow rate to 1 mL/min (instead of 0.6 mL/min).
- Condition B: HPLC mobile phase modification.
- Solvent A = H20 + 0.01% TFA (instead of 0.1% TFA).
- Solvent B = ACN + 0.01% TFA (instead of 0.1% TFA).

Two other conditions were listed for the TLC method:

- Condition A: mobile phase with 100% of Methanol solution.
- Condition B: mobile phase with 100% of 1M ammonium acetate.

For each condition, we evaluated the retention time (HPLC), the retardation factor (TLC) and the RCP.

#### 4.4.6. Quantification Limit (LoQ)

These limits were calculated by the signal-to-noise ratio approach. We decided to find these values for the impurities assay, because they corresponded to the lowest fraction in the RCP determination (impurities < 3%). We compared the signal obtained with samples containing known low concentrations of the analyte to the signal obtained with blanks. Then, we determined the lowest concentration at which the substance can be reliably quantified. A signal-to-noise ratio of 10:1 was used to assess the quantification limit. 

#### 4.4.7. Range

The applicable measurement range was defined based on the assessment of linearity and limit of quantification. The upper activity limit is determined by the intrinsic characteristics of detector response, which is known to be linear provided that deadtime does not exceed 5% [15].

The applicable radioactivity range for the samples of ^177^Lu-PSMA-1 was calculated thanks to the product specifications.

## 5. Conclusions

We developed and validated HPLC as well as a TLC analytical method to assess the radiochemical purity of the radiotracer ^177^Lu-PSMA-1. Indeed, the absence of monographs in the European pharmacopoeia requires us to validate the analytical methods in order to use this new radiopharmaceutical in clinical routine. For this purpose, we used the recent EANM recommendations [15], the European Pharmacopoeia monograph guidelines [17] for radiopharmaceuticals and the Q2 (R1) ICH guidelines [26] to validate these analytical methods. The results obtained in this study allowed for the validation of precise, accurate, linear, robust and sensitive HPLC and TLC methods and can be implemented in routine quality control. This allows us to safely release a production batch of ^177^Lu-PSMA-1, a new radiopharmaceutical useful for treatment of patients with hormone-resistant and metastatic prostate cancer.

## Figures and Tables

**Table 1:** Retention time ^117^Lu-PSMA, retention time for free ^177^Lu impurity, peak area, tailing factor, plate count and resolution in HPLC.

	^177^Lu-PSMA-1 Retention Time (min)	Free ^177^Lu Retention Time (min)	Peak Area: Counts per Second (cps)	Tailing Factor (TF)	Plate Count (N)	Resolution (Rs)
Mean	17.32	2.32	108 895	1.25	6725.70	17.73
Standard deviation	0.35	0.35	9270	0.08	153.60	0.44
Variation coefficient (%)	0.05	0.38	8.51	1.18	2.28	2.50

**Table 2:** Retardation factor and resolution in TLC.

	^177^Lu-PSMA-1 Retardation Factor	Free ^177^Lu Retardation Factor	Resolution (Rs)
Mean	0.930	0.142	3.060
Standard deviation	0.020	0.001	0.400
Variation coefficient (%)	1.730	0.700	13.600

**Table 3:** Accuracy with % recovery in HPLC.

% ^177^Lu-PSMA-1	Recovered RCP	Theoretical RCP	% Recovery
100	99.72	99.72	100.00
90	90.29	89.75	100.61
80	80.57	79.78	101.00
70	70.81	69.80	101.44
60	60.83	59.83	101.67

**Table 4:** Accuracy with % recovery in TLC.

% ^177^Lu-PSMA-1	Recovered RCP	Theoretical RCP	% Recovery
100	99.42	99.42	100.00
90	89.75	89.47	100.30
80	80.07	79.53	100.67
70	69.68	69.59	100.12
60	58.74	59.65	98.47

**Table 5:** Precision/repeatability and intermediate precision in HPLC.

**HPLC Method (*n* = 6)**	**Radiochemical Purity (RCP) ± SD (%)**	**RSD (%)**	**Retention Time** **Mean ± SD (min)**	**RSD (%)**
Repeatability	98.52 ± 0.15	0.15	17.53 ± 0.04	0.24
**HPLC Method** **(*n* = 9)**	**RCP (%)** **Mean ± SD**	**RSD (%)**	**Retention Time** **Mean ± SD (min)**	**RSD (%)**
Intermediate precision: 3 series (Analyst 1 + Analyst 2 + Analyst 3)	98.79 ± 0.72	0.73	17.31 ± 1.18	0.12

**Table 6:** Precision/repeatability and intermediate precision in TLC.

**TLC Method (*n* = 6)**	**Radiochemical Purity (RCP) ± SD (%)**	**RSD (%)**	**Retardation Factor** **Mean ± SD**	**RSD (%)**
Repeatability	99.84 ± 0.03	0.03	0.93 ± 0.01	1.26
**TLC Method** **(*n* = 9)**	**RCP (%)** **Mean ± SD**	**RSD (%)**	**Retardation Time** **Mean ± SD (min)**	**RSD (%)**
Intermediate precision: 3 series (Analyst 1 + Analyst 2 + Analyst 3)	99.12 ± 0.89	0.90	0.92 ± 0.03	2.99

**Table 7:** Summary table of criteria and their results.

Test	Acceptance Criteria	HPLC Results	TLC Results
% Recovery	90% < % recovery < 110%	100.00% to 101.67%	98.47–100.67%
Specificity	Resolution factor (Rs) > 2	17.73	3.060
Linearity	R^2^ > 0.99	0.9978	0.9997
Intermediate Precision	RCP: RSD < 2%	0.73	0.90
	Retardation time: RSD < 2%	0.12	2.99
Repeatability	RCP: RSD < 2%	0.15	0.03
	Retardation time: RSD < 2%	0.24	1.26
Robustness	RSD < 2%	0.06% (condition A) 0.30% (condition B)	0.51
Quantification limit (LoQ)	Signal-to-noise ratio ≥ 10	33.8 kBq (12.7:1)	61.8 kBq (10.8:1)
Range	Reported value	2.5 to 500 MBq/mL	

## Data Availability

Data is contained within the article and Supplementary Materials.

## Supplementary Materials

The following supporting information can be downloaded at: https://www.mdpi.com/article/10.3390/ph15050522/s1, Figure S1: Linearity between the concentration of the standard and the peak area; Figure S2: Linearity with the correlation of the recovered RCP and the theoretical RCP in HPLC; Figure S3: Linearity with the correlation of the recovered RCP and the theoretical RCP in TLC; Table S1: Linearity of 175Lu-PSMA I/T measurements.
