# Supplementary material for: Development and Validation of Analytical Methods for Radiochemical Purity of 177Lu-PSMA-1
Source: Pharmaceuticals (Basel). 2022 Apr 24;15(5):522. doi: 10.3390/ph15050522 (PMC9143309; doi:10.3390/ph15050522)

Supplementary Materials:

For each concentration injected in HPLC, the mean area under the curve values as well as the mean retention times were collected. The results are detailed in **Table S1**.

Table S1. Linearity of ^{175}Lu-PSMA I/T measurements

Concentration ^{175}Lu-PSMA I/T (µg/mL)	Integrated peak areas Mean ± SD (mAU.s)	RSD (%)	Retention time Mean ± SD (min)	RSD (%)
500	5286.67 ± 53.379	1.01	17.30 ± 0.002	0.34
300	3048.33 ± 36.226	1.19	17.27 ± 0.001	0.15
150	1711.67 ± 28.148	1.64	17.28 ± 0.003	0.46
75	884.67 ± 10.504	1.19	17.32 ± 0.005	0.72
37.5	382.00 ± 7.937	2.08	17.43 ± 0,001	0.20
15	129.33 ± 3.055	2.36	17.43 ± 0.001	0.11

The straight linear regression equation permitted to calculate the linear correlation coefficient which was 0.9981 (**Figure S1**). The linearity between the concentration of the standard and the peak area was demonstrated.

Figure S1. Linearity between the concentration of the standard and the peak area

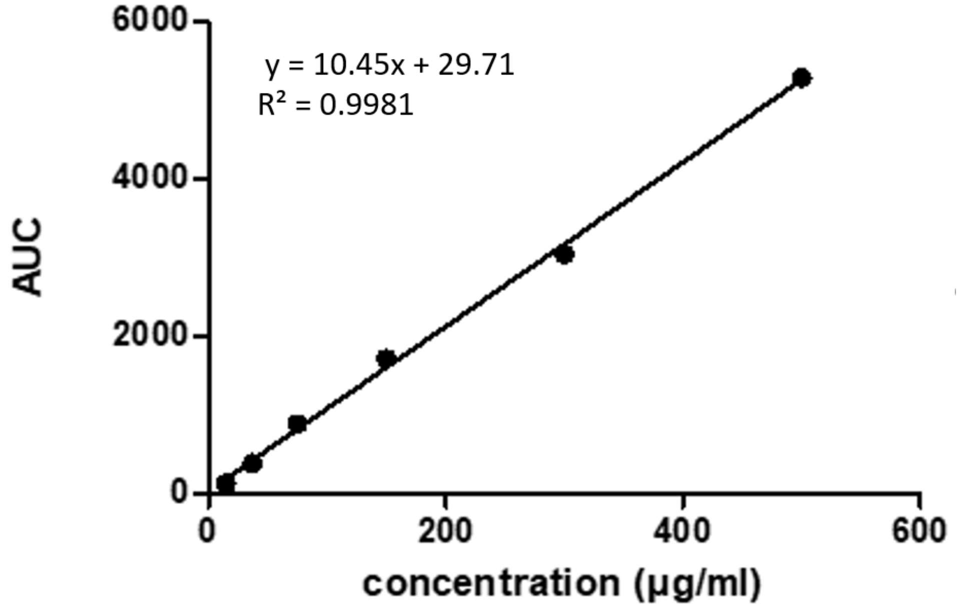

The linearity can also be evaluated by the correlation of the recovered RCP and the theoretical RCP of ^{177}Lu-PSMA-1 by retrieving the accuracy data.
Below, the calibration curves between the recovered RCP and the theoretical RCP were demonstrated for HPLC and TLC methods (**Figures S2 and S3**).

Figure S2: Linearity with the correlation of the recovered RCP and the theoretical RCP in **HPLC**

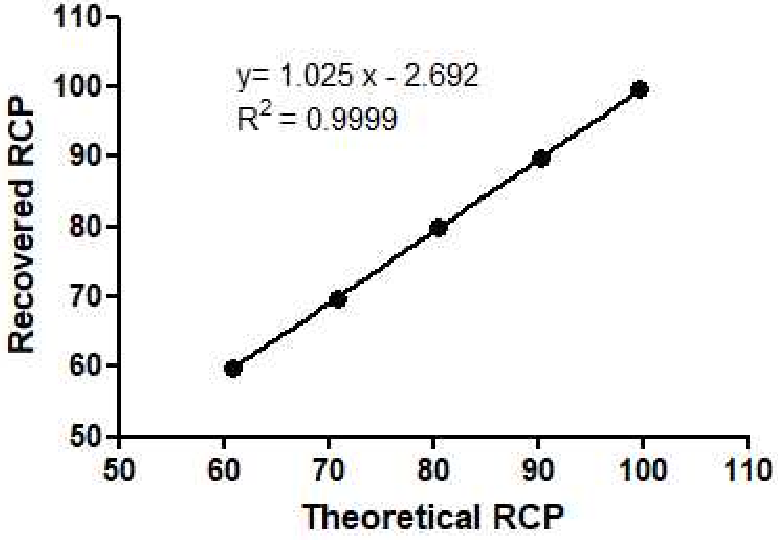

Figure S3: Linearity with the correlation of the recovered RCP and the theoretical RCP in **TLC**

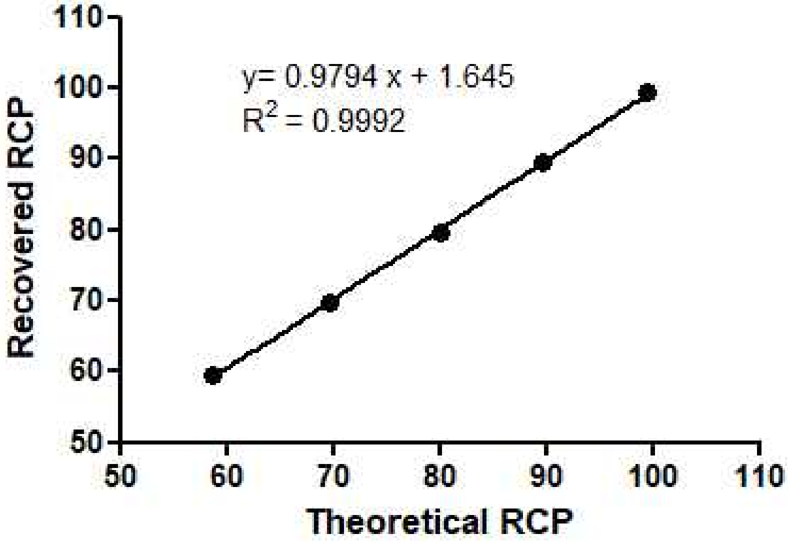

Supplement: Supplementary file 1 [file pharmaceuticals-15-00522-s001.zip › pharmaceuticals-1687296-supplementary.pdf]
